# Biofeedback-based Digital Games and Well-being in Childhood: A Systematic Review

**DOI:** 10.1007/s10802-025-01387-x

**Published:** 2025-11-20

**Authors:** Morgan D. Darabi, Sara P. Silber, Rebecca Slotkin, Alyssa L. Peechatka

**Affiliations:** 1 Department of Psychology, The Pennsylvania State University, 247 Moore Building, University Park, PA 16802 United States of America; 2 Department of Pediatrics, University of Miami, Miami, FL 33136 USA; 3 Department of Psychology, The Catholic University of America, Washington, DC 20064 USA; 4 Division of Psychology, Boston Children’s Hospital, Boston, MA USA

## Abstract

Biofeedback-based digital games offer an engaging solution to the burgeoning youth mental health crisis. This review identified and evaluated empirical evidence of biofeedback-based digital games in the treatment of youth mental health challenges and the promotion of youth well-being. A systematic search of four electronic databases (CINAHL, PsycInfo, PubMed Central, and Web of Science) was conducted from the publication’s year of inception. We identified 604 unique studies, 16 of which were included in the review based on eligibility criteria. The results of 7 studies were deemed generalizable due to their design. Participants in these 7 studies played *Dojo*,* emWave*,* Mightier*, *RAGE-Control*, or *Wild Divine*, all of which utilized a form of cardiac biofeedback. These games proved effective in reducing internalizing symptoms, externalizing behaviors, and general psychopathology, especially within clinical populations. In addition, there is promising evidence of their ability to promote emotion regulation skills as a universal intervention. Future directions include further evaluation of biofeedback-based digital games in school-based settings, as an interim treatment for waitlisted families in outpatient settings, as an add-on to existing evidence-based treatments, and of the cost-benefits to promote insurance coverage of biofeedback-based digital interventions.

## Introduction

Poor mental health in childhood is the leading cause of negative life outcomes (Perou et al., 2013). Traditional mental health care systems, which often rely on 1:1 service delivery with a mental health professional through weekly fee-for-service appointments, fail to meet the need of our nation’s youth; about half of the children with treatable mental health conditions do not receive sufficient care (Whitney & Peterson, 2019). Digital health interventions, such as biofeedback-based digital games, offer an engaging, scalable, and accessible opportunity to treat mental health disorders as well as prevent the onset of disorders through the promotion of well-being in childhood. However, the integration of biofeedback mechanisms into digital games is a relatively new strategy to target youth mental health concerns, and the current state of evidence remains unclear. This review aims to uncover the digital games and biofeedback mechanisms that have been used, the domains of mental health and well-being that have been targeted, characteristics of study participants, and the quality of evidence with a goal of providing guidance on next steps in the development, evaluation, and implementation of biofeedback-based digital games.

### Mental Health and Well-being in Childhood

The majority of mental health conditions begin before the age of 24 (75%; Jones, 2013). Globally, approximately 14% of youth aged 10 to 19 years old have a diagnosed mental health disorder, with rates soaring to 20% or higher in some countries (World Health Organization, 2024a). In the United States (U.S.), 1 in 6 children aged 6 to 17 years old have a diagnosed mental health disorder (Whitney & Peterson, 2019), and suicide is the third leading cause of death among adolescents (World Health Organization, 2024c). In 2019, almost 20% of high school students reported seriously considering suicide in the past year (Bitsko, 2022). The prevalence of youth mental health disorders has increased over time (Tkacz & Brady, 2021), with surges evident during and after the COVID-19 pandemic (Lebrun-Harris et al., 2022; Racine et al., 2021). Disruptions in schooling, social isolation, and increased exposure to familial stressors had profound consequences on youth well-being, contributing to elevated levels of psychological distress (Hawrilenko et al., 2021; Power et al., 2020). In particular, researchers identified a surge in rates of childhood anxiety and depression, with estimates increasing from 12% to 19% and 9% to 24%, respectively (Burdzovic Andreas & Brunborg, 2017; Racine et al., 2021; Tiirikainen et al., 2019).

As a result, several countries have declared a youth mental health crisis or state of emergency, emphasizing an urgent and indisputable need for greater investment in efforts that promote youth mental health and well-being (American Academy of Pediatrics, 2021; Benton et al., 2024; World Health Organization, 2024b). Experts are calling for coordinated action across health, education, and social service systems to address these growing needs and create supportive environments that foster well-being for children and adolescents (Benton et al., 2022). The long-term impact of neglecting these needs could further exacerbate inequities in health and education (Idele & Banati, 2021).

### Access To and Use of Mental Health Services in Childhood

Despite the growing prevalence of youth mental health challenges, only about half of children and adolescents with a diagnosed mental health disorder receive related services and treatment (51%; Whitney & Peterson, 2019). Many families face significant barriers to accessing mental health care for their children, and a litany of factors limit comfort engaging with mental health services (Anderson et al., 2017). In particular, children in families of low-income, children living in rural communities, and children of color are less likely to access mental health services (Marrast et al., 2016; Morales et al., 2020; Steele et al., 2007; Ziller et al., 2010).

Over a third of children in the U.S. live within a family of low-income (38%), and about a fifth of children in the U.S. are living below the poverty line (17%; National Center for Children in Poverty, 2021). Despite evidence that children in families of low-income experience greater levels of psychopathology (Peverill et al., 2021), the high cost of mental health care, combined with lower levels of insurance coverage, often prohibit families from utilizing traditional mental health services (weekly fee-for-service appointments in outpatient or community settings; Salloum et al., 2016). Despite the expansion of insurance coverage under the Affordable Care Act, out-of-pocket expenses push services out of reach for many families (Coombs et al., 2021). On average, weekly outpatient therapy in the U.S. ranges from $100 to $200 per session (Barron, 2021). In addition, logistical barriers, such as a lack of transportation, time, or community providers, prevent access to mental health care. In many families, especially families of low-income, caregivers working full-time or multiple jobs do not have the ability to bring their child to a weekly therapy appointment, and many do not have access to transportation to reach the therapy office or clinic (Reardon et al., 2017). In addition, a shortage of community providers limits the accessibility of mental health services, especially for children living within rural communities (Palomin et al., 2023). Even when community providers are available, there is often an underutilization of mental health services within rural communities (Andrilla et al., 2018; Kepley & Streeter, 2018).

Demographic factors, such as race and ethnicity, are also significant predictors of treatment access; children with minoritized racial and ethnic identities are significantly less likely to have access to or engage with mental health services (Marrast et al., 2016). Negative experiences with and stigma around utilizing mental health care have a profound impact on the utilization in childhood and adolescence. Historically, people of color have faced mistreatment and racism from healthcare professionals, leading to a mistrust of healthcare systems (Whaley, 2001). Children of color who do seek services are also less likely to receive diagnoses and appropriate service recommendations, further increasing tension and disparities in the receipt of services (Liang et al., 2016). Moreover, families for whom English is not a proficient language are much less likely to attain services for their child, even if their child does speak English proficiently (Ohtani et al., 2015). This issue is of increasing concern as the number of individuals in the U.S. who do not speak English proficiently continues to rise annually (U.S. Census Bureau, 2020).

Despite the increased prevalence of campaigns to improve public knowledge and understanding of mental health challenges (Pescosolido et al., 2021), stigma around the utilization of mental health services continues to prevent families from seeking care for their children (Heflinger & Hinshaw, 2010). Families often face stigma that youth mental health challenges are due to poor parenting, which can lead to a belief that such challenges are not psychological in nature or that treatment will result in familial blame or shame (Drent et al., 2022). Additionally, youth report a fear of negative responses from their peers and family as a primary factor that prevents them from seeking services (Chandra & Minkovitz, 2007). Though mental health providers and researchers have continuously investigated methods to decrease stigma and increase service usage, it remains a significant barrier for many youth and their families (DeLuca, 2020; Heflinger & Hinshaw, 2010).

A lack of access to and utilization of mental health services often results in poor outcomes for youth (Dell’Osso & Altamura, 2010; Wang et al., 2005). Untreated mental illness during childhood and adolescence can lead to poor social functioning, worse educational outcomes, and, in extreme cases, premature death due to untreated suicidality (McGorry & Mei, 2020). The increased demand for services since the COVID-19 pandemic has led to extensive wait times and prolonged distress for youth (Benton et al., 2021). This can especially impact those living in small or rural communities that might only have one provider or clinic to serve the entire community (Mongelli, 2020). Substantial and systemic change is needed to ensure equity in access to care and best support all youth in the U.S. (Moreno et al., 2020).

### Dital Health Interventions

Digital health interventions are increasingly explored as a mechanism to address the growing need for youth mental health support (Lattie et al., 2022), and The White House (2024) identified understanding and leveraging digital mental health interventions as a research priority. Digital health interventions can increase equitable access to care by eliminating or reducing many barriers that exist for rural, low-income, and other typically underserved communities (Schueller et al., 2019). Specifically, well designed digital mental health tools can be accessed in the home, at any time, without stigma, and often in an individual’s preferred language (Bucci et al., 2019; Lattie et al., 2022). They do not require physical proximity to a trained mental health professional, which allows them to be provided at a fraction of the cost when compared to mental health services that rely on a 1:1 service delivery model (Lattie et al., 2022). Researchers have identified digital health interventions as a promising strategy in supplementing and supplanting traditional mental health care for youth (Lehtimaki et al., 2021). However, few existing digital interventions are empirically supported (Lehtimaki et al., 2021), and many contain barriers to engagement that reduce sustained interaction (Borghouts, 2021).

### Biofeedback and Game-based Interventions

Biofeedback is a self-regulation technique in which individuals learn to control bodily processes (Frank et al., 2010). Biofeedback based tools offer an opportunity for users to control seemingly involuntary physiological processes by exploring the relationship between bodily states and their actions, the environment, or other variables within the framework of the tool (Austad & Gendron, 2018; Schwartz, 2017). During biofeedback training, technological equipment is typically used to convert physiological signals into visual or auditory cues (Frank et al., 2010). The most common types of biofeedback include cardiac, which measures heart rate (HR) or heart rate variability (HRV), respiratory, which measures breathing activity or oxygen saturation, galvanic, which measures skin conductance levels (SCL) or electrodermal activity (Windthorst et al., 2015) and neurofeedback or electroencephalogram (EEG), which measures electrical activity within the brain (Marzbani et al., 2016).

When considering biofeedback tools as mental health interventions, they function to make abstract concepts, such as emotional states or regulation, more concrete and tangible (Sadka & Antle, 2022). Most commonly, researchers have explored the ability of biofeedback to help individuals manage stress (Kennedy & Parker, 2019) and anxiety (Alneyadi et al., 2021). In particular, Yu and colleagues (2018) identified biofeedback as a promising stress management strategy but recommended new interaction designs to promote accessibility, engagement, and user experience. A recent meta-analysis also identified associations between HRV biofeedback and reductions in self-reported stress and anxiety but recommended additional controlled studies to bolster empirical support (Goessl et al., 2017).

The ability to translate abstract concepts into experiential learning is particularly helpful to youth at varying developmental stages as it does not rely on cognitive or linguistic capacities and capitalizes on constructivist theories of learning (Kolb, 2014). However, traditional biofeedback that does not include gamified components can be monotonous for youth, resulting in lower levels of engagement and premature treatment dropout (Jercic & Sundstedt, 2019). Layering a gamified component onto biofeedback-based tools creates a space where youth are intrinsically motivated to take on challenging tasks (Jercic & Sundstedt, 2019) and fosters a safe space for failure and iteration-based learning while creating a powerful sense of autonomy for the user (Tsay et al., 2020). From a practical perspective, gamification increases enjoyment and reward involved in the task, potentially reducing high dropout rates due to the monotony of typical biofeedback interventions (Richter et al., 2015).

## Current Study

Given the soaring rates of youth mental health disorders and the lack of service utilization, there is an urgent and indisputable need for accessible interventions (American Academy of Pediatrics, 2021; Lebrun-Harris et al., 2022; Whitney & Peterson, 2019). Digital health interventions, such as biofeedback-based digital games, offer promise as an engaging, scalable, and accessible tool (Austad & Gendron, 2018; Jercic & Sundstedt, 2019; Lattie et al., 2022). However, the current state of evidence for biofeedback-based digital games in the promotion of youth well-being is unknown. We aimed to identify and evaluate existing empirical evidence to better understand the type of digital games and accessible biofeedback mechanisms that have been used, the domains of mental health and well-being that have been targeted, characteristics of study participants, and the quality of evidence for the treatment and prevention of youth mental health disorders in order to provide guidance on next steps for the development, evaluation, and implementation of biofeedback-based digital games.

## Method

### Eligibility Criteria

This review included peer-reviewed empirical studies that incorporated a biofeedback mechanism with both digital and playful components to address any domain of youth mental health or well-being. Biofeedback was defined as any intervention that used a potentially scalable measure of a bodily process (e.g., HR, HRV, SCL) to provide feedback. In order to qualify as digital and playful, the intervention had to include gamification via digital technology (e.g., play via a tablet, mobile device, or computer). At least one outcome measure of the study had to address a domain of mental health or well-being. For example, studies addressing symptomology of a mental health disorder (e.g., anxiety or depressive symptoms) as well as studies aiming to foster skills that protect against the development of mental health disorders or promote well-being (e.g., emotion regulation, relaxation) were included in this review. Lastly, studies were only included if they focused on a pediatric population. Studies were included with participants over age 18 as long as the mean age of the sample was less than or equal to 17 years old. There was no enforcement of publication date or publication status restrictions. Studies not available in English and studies that included measures of brain wave activity, such as neurofeedback or EEG biofeedback, were excluded from review, as the reliance on expensive equipment and trained technicians does not meet the stated goal of evaluating scalable and accessible youth mental health care options.

### Information Sources and Search Terms

Studies were identified through a systematic search of four electronic databases, scanning reference lists of relevant articles, and examining studies included within identified review papers. This search included PubMed (1966-Present), PsycInfo (1927-Present), CINAHL (1937-Present), and Web of Science (1900-Present). The last search was run on May 27, 2023. The following search terms were used for all databases: Biofeedback; Biofeedback Training; Psychology; Augmented Reality; Children’s Games; Computer Applications; Computer Games; Digital Game-Based Learning; Digital Gaming; Digital Health; Digital Technology; Exergam*; Game*; Games, Experimental; Gamif*; Gaming; Role Playing Games; Simulation Games; Video game*; Videogame*; Virtual Environment; Virtual Reality; Virtual Reality Exposure Therapy; Visual Feedback; Wearable Devices; Adolesc*; Adolescent Behavior; Adolescent Development; Adolescent Health; Adolescent Health Services; Adolescent Medicine; Adolescent Psychiatry; Adolescent Psychology; Child Behavior; Child Development; Child Health; Child Health Services; Child Psychiatry; Child Psychology; Child, Preschool; Child*; Childhood Development; Early Adolescence; Infant Behavior; Infant Development; Infant Health; Infant*; Paediatric*; Pediatric Care; Pediatric*; Preschool*; Psychology, Adolescent; Psychology, Child; Teen*; Toddler*; Youth*. The search was developed and conducted by the first author in consultation with a university librarian who had expertise in this literature base. See Appendix A for the specific electronic search strings applied in each database.

### Study Selection

Eligibility assessment was performed in three rounds by the first, second, and fourth authors in a standardized manner. In the first round, each of the authors was assigned to screen two thirds of the article titles for eligibility, such that each title was screened by two authors. A similar process was followed to screen the abstracts of the remaining studies. In cases of disagreement between the two assigned reviewers in the first two rounds, the study would be included in the next round. Lastly, the full texts of all remaining articles were screened for eligibility. In this third round, disagreements were discussed and resolved through consensus.

### Data Collection Process

The first author developed a data extraction sheet, which was pilot tested with the second author and multiple research assistants using six sample papers that were excluded due to participant age. The sheet was refined after each sample paper was coded to ensure clarity and ease of use. Each included paper was then independently coded by two research assistants who were trained in how to extract data using this sheet over multiple weeks by the first and second authors. Disagreements in abstraction were resolved by the first author. The data extraction sheet included: (1) Article characteristics (Year of publication and peer review status), (2) Sample characteristics (Sample size, geographic location, setting, age, gender, race, and ethnicity), (3) Details of intervention (Biofeedback mechanism, experimental conditions, digital game component), and (4) Target of intervention (Mental health disorder and/or domain of well-being). If a paper did not include this information, the second author contacted the corresponding author of each paper via email to request additional information.

### Appraisal of Individual Studies

The guidelines outlined by Purssell and McCrae (2020) were followed to critically appraise the strengths and weaknesses of each study. The first and third author each reviewed the following for half of the included studies such that each study was reviewed once: (1) Hierarchies of evidence (Type of study design), (2) Quality of reporting and methodology (Clear description of aims and hypotheses, randomization, blinding, lack of differences at baseline, effect size, generalizability of results, consideration of outcomes, risk-benefit analysis), and (3) Risk of bias using the Cochrane Risk of Bias Tool-2 for randomized studies (Bias due to randomization, deviation from intended intervention, missing data, outcome measurement, selection of reported results; Higgins et al., 2019; Sterne et al., 2019) and ROBINS-I for non-randomized studies (Bias due to confounding, classification of intervention, deviation from intended intervention, missing data, outcome measurement, selection of reported results; Sterne et al., 2016).

## Results

### Study Selection

**Fig. 1:**
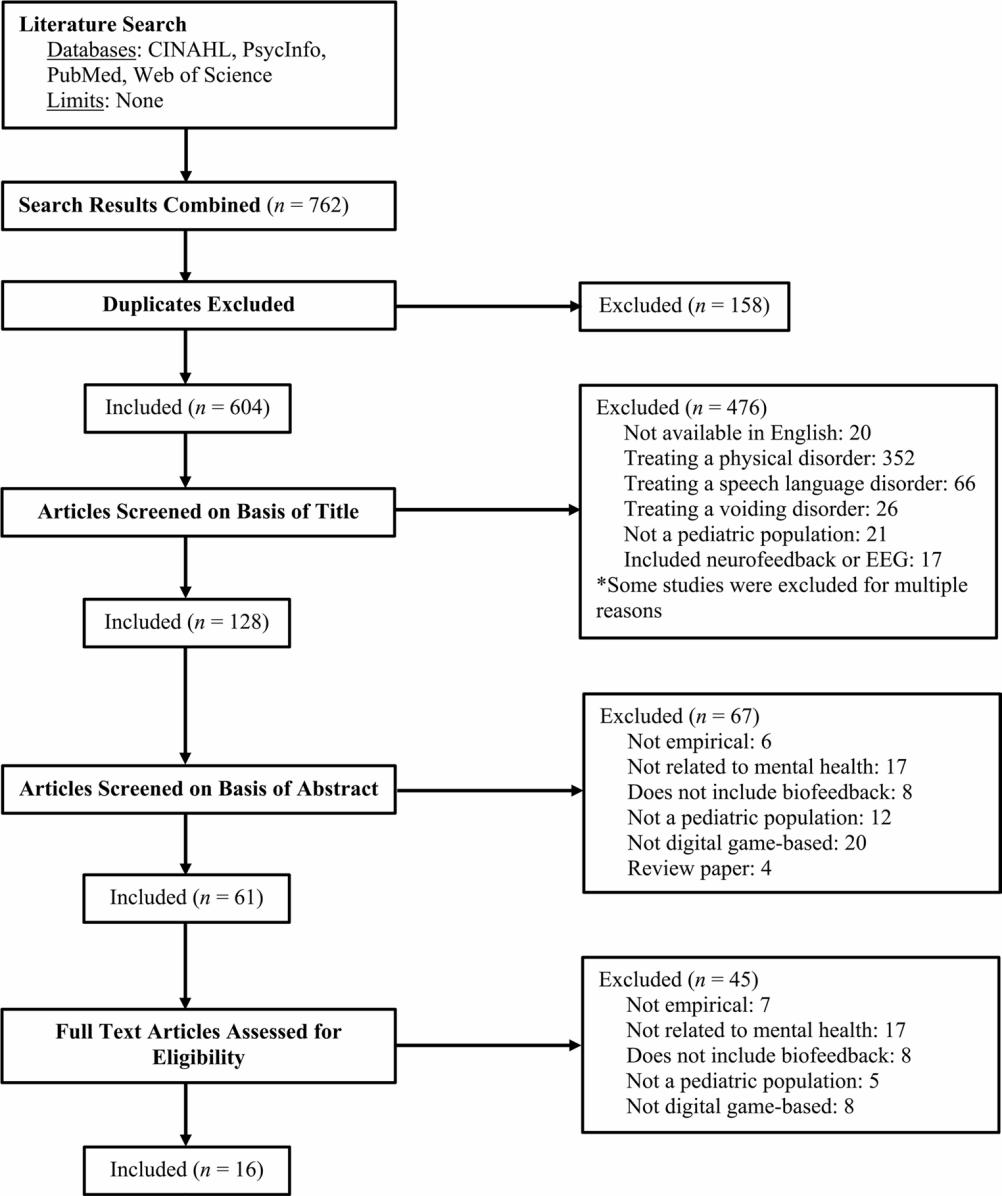
Flow diagram of study selection

The systematic search of four electronic databases (CINAHL, PsycInfo, PubMed Central, and Web of Science) resulted in the identification of 762 studies. The authors followed PRISMA guidelines to screen articles for inclusion (Moher et al., 2009). Duplicate studies were removed, which resulted in 604 unique studies. After screening on the basis of title, 467 studies were excluded from this review due to: (1) Lack of availability in the English language (*N* = 20); (2) Targeting of a physical disorder (*N* = 352), speech language disorder (*N* = 66), or voiding disorder (*N* = 26); (3) Non-pediatric population (*N* = 21); and/or (4) Inclusion of neurofeedback or EEG biofeedback (*N* = 17). Of note, some studies were excluded for multiple reasons. The abstracts of the remaining 128 studies were then screened for eligibility. This resulted in 67 studies being excluded from review due to: (1) Lack of peer-review and/or empirical evidence (*N* = 6); (2) Lack of related outcomes (*N* = 17); (3) Lack of biofeedback use (*N* = 8); (4) Non-pediatric population (*N* = 12); or (4) Lack of a digital game component (*N* = 20). In addition, four review papers were removed after reviewing the included studies within them, none of which met the study criteria. Then, the full texts of the remaining 61 studies were reviewed for inclusion, which resulted in 45 studies being excluded from review due to: (1) Lack of peer-review and/or empirical evidence (*N* = 7); (2) Lack of related outcomes (*N* = 17); (3) Lack of biofeedback use (*N* = 8); (4) Non-pediatric population (*N* = 5); or (5) Lack of a digital game component (*N* = 8). This resulted in the inclusion of 16 studies. See Fig. 1 for the PRISMA flow chart outlining this process.

### Study Characteristics

#### Participants

Sample sizes of the 16 included studies ranged from 8 participants to 1,045 participants (M = 98.6; Md = 30.5). Participants ranged in age from 3 to 30 years old, with average sample ages ranging from 9 to 17 years old. In terms of gender, samples ranged from 13% to 100% female. Of note, only four studies included racial and/or ethnic identities of participants (Burkhart et al., 2018; Ducharme et al., 2021; Knox et al., 2011; Scholten et al., 2016).

The majority of studies that reported the geographic location of the sample were conducted in the U.S. (Burkhart et al., 2018; Ducharme et al., 2021; Fish, 2018; Kahn et al., 2013; Knox et al., 2011; Mannweiler et al., 2023; Rush et al., 2017; Wintner et al., 2022; Woodberry et al., 2021). Four studies were conducted in the Netherlands (Bossenbroek et al., 2020; Scholten et al., 2016; Schuurmans et al., 2015, 2018), and one study was conducted in each Australia (Amon & Campbell, 2008) and New Zealand (Thabrew et al., 2021). Lastly, five studies reported collecting their data within a hospital setting (Burkhart et al., 2018; Ducharme et al., 2021; Kahn et al., 2013; Thabrew et al., 2021; Woodberry et al., 2021). Three studies were conducted in school settings (Bossenbroek et al., 2020; Rush et al., 2017; Scholten et al., 2016), and an additional three studies were conducted in residential care settings (Jaramillo-Quintanar et al., 2020; Schuurmans et al., 2015, 2018). Two studies were conducted on university campuses (Fish, 2018; Knox et al., 2011). The final two studies were conducted in a community setting (Mannweiler et al., 2023) and online (Wintner et al., 2022). See Table 1.

**Table 1:** Participant characteristics

	*N*	Age	Gender	Geographic Location	Setting
Amon & Campbell, 2008	36	NP (*M* = 9)	38%	Australia	NP
Bossenbroek et al., 2020	8	12–17 (*M* = 15)	13%	Netherlands	School
Burkhart et al., 2018	30	11–21 (*M* = 16)	60%	United States	Hospital
Ducharme et al., 2021	40	10–17 (*M* = 13)	28%	United States	Hospital
Fish, 2018	10	NP (*M* = NP)	NP	United States	University
Jaramillo-Quintanar et al., 2020	29	6–12 (*M* = 9)	NP	NP	Residential Care
Kahn et al., 2013	37	9–17 (*M* = NP)	NP	United States	Hospital
Knox et al., 2011	24	9–17 (*M* = 13)	38%	United States	University
Mannweiler et al., 2023	72	7–12 (*M* = NP)	100%	United States	Community Setting
Rush et al., 2017	31	8–13 (*M* = 10)	13%	United States	School
Scholten et al., 2016	138	11–15 (*M* = 13)	65%	Netherlands	School
Schuurmans et al., 2015	8	NP (*M* = 14)	38%	Netherlands	Residential Care
Schuurmans et al., 2018	37	NP (*M* = 14)	16%	Netherlands	Residential Care
Thabrew et al., 2021	24	11–17 (*M* = 14)	63%	New Zealand	Hospital
Wintner at al., 2022	1045	3–19 (*M* = 9)	28%	United States	Online
Woodberry et al., 2021	8	12–30 (*M* = 17)	27%	United States	Hospital

*N* Sample size, *NP *Not provided. Gender is represented as the percentage of the sample that identified as female

#### Digital Games and Biofeedback Mechanisms

Ten unique digital games were played across the sixteen studies. Four games were played via a computer, three games were played via a tablet or mobile device, two games were played via virtual reality (VR), and one game was played with a screen and handheld controller. Most studies incorporated HR or HRV biofeedback. Respiratory and SCL biofeedback were also used alone or in addition to HR or HRV biofeedback. See Table 2 for an overview of biofeedback mechanisms.

**Table 2:** Overview of study designs and outcomes by targets of interventions

		**Internalizing Symptoms**			
	***N***	**Study** **Design**	**Biofeedback Mechanism**	**Outcome** **Measure**	**Effect** **Size**
Bossenbroek et al., 2020	8	Case Study	Respiratory	Anxiety*	Small (*d* = − 0.29)
Burkhart et al., 2018	30	Pilot Study	HRV	SUDs*	Large (*d* = 1.85)
Ducharme et al., 2021	40	Blinded RCT	HR	Anger	NP
Fish, 2018	10	Pilot Study	SCL	Stress	NA^a^
Jaramillo-Quintanar et al., 2020	29	Feasibility Trial	Respiratory/HR	Relaxation	NP
Kahn et al., 2013	37	Controlled Trial	HR	State Anger*	NP
				Trait Anger*	NP
Knox et al., 2011	24	RCT	HRV/SCL	Anxiety*	Large (η² =0.51)
				Depression*	Large (η² = 0.45)
				State vs. Trait Anxiety*	Large (η² = 0.15)
Mannweiler et al., 2023	72	RCT	HR	Parent Stress*	Large (η² = 0.23)
Scholten et al., 2016	138	RCT	HRV	Anxiety	Negligible (*d =* 0.08)
Schuurmans et al., 2015	8	Pilot Study	HRV	Anxiety*	Large (η² = 0.36 − 0.70)
Schuurmans et al., 2018	37	RCT	HRV	Anxiety*	Large (η² = 0.14 − 0.16)
Thabrew et al., 2021	24	Open Trial	HR	Anxiety*	Medium (*d* = 0.60)
Woodberry et al., 2021	8	Feasibility Trial	HR	Perceived Stress*	Small (*g* = − 0.26)
		**Externalizing Behaviors**			
	***N***	**Study** **Design**	**Biofeedback** **Mechanism**	**Outcome** **Measure**	**Effect** **Size**
Amon & Campbell, 2008	36	Controlled Trial	HR/SCL	ADHD Symptoms*	Large (η² = 0.57)
Bossenbroek et al., 2020	8	Case Study	Respiratory	Disruptive Behaviors	Small (*d* = − 0.16)
Ducharme et al., 2021	40	Blinded RCT	HR	Overt Aggression*	Large (*r* =.55)
				Oppositional Behaviors*	Medium (*r* =.48)
Rush et al., 2017	31	Blinded Controlled Trial	HR/HRV/Respiratory	On-task Behaviors	Medium (*d* = − 0.71)
				Off-task Behaviors*	Large (*d* = 0.99)
Schuurmans et al., 2015	8	Pilot Study	HRV	Externalizing Behaviors*^b^	Negligible to Large (η² = 0.00 − 0.25)
Schuurmans et al., 2018	37	RCT	HRV	Externalizing Behaviors*^b^	Small to Medium (η² = 0.06 − 0.11)
		**Emotion Regulation**			
	***N***	**Study** **Design**	**Biofeedback** **Mechanism**	**Outcome** **Measure**	**Effect** **Size**
Mannweiler et al., 2023	72	RCT	HR	Emotion Regulation*	Large (η² = 0.42 − 0.43)
Wintner at al., 2022	1045	Observational Study	HR	Emotion Regulation*	Small (*r* =.10)
		**General** ** Psychopathology/Functioning**			
	***N***	**Study** **Design**	**Biofeedback** **Mechanism**	**Outcome** **Measure**	**Effect** **Size**
Amon & Campbell, 2008	36	Controlled Trial	HR/SCL	Functioning*	Large (η² = 0.37)
Ducharme et al., 2021	40	Blinded RCT	HR	Psychopathology*	Large (*r* =.51)
Mannweiler et al., 2023	72	RCT	HR	Behaviors and Feelings	Small (*r* =.22)
Woodberry et al., 2021	8	Feasibility Trial	HR	Functioning*^c^	Small to Medium (|*g| =* 0.29 − 0.57)

*N* Sample size, *NP *Not provided, *NA* Not applicable, *RCT* Randomized Controlled Trial, *SUDs* Subjective Units of Distress, *ADHD* Attention-deficit/hyperactivity disorder, *HR* Heart rate, *HRV* Heart Rate Variability, *SCL* Skin Conductance Levels. * indicates statistically significant change or differences between groups. ^a^ indicates not applicable due to qualitative analysis. ^b^ indicates significant change in self-reported externalizing behavior and non-significant change in mentor-reported externalizing behavior. ^c^ indicates statistically significant improvement in social functioning subscale only

Three studies tasked participants with playing *Wild Divine* via a computer (Amon & Campbell, 2008; Burkhart et al., 2018; Knox et al., 2011). Depending on the version of the game, children either wore biofeedback sensors on their fingers (Amon & Campbell, 2008; Knox et al., 2011) or earlobes (Burkhart et al., 2018) to provide feedback via HR (Amon & Campbell, 2008), HRV (Burkhart et al., 2018; Knox et al., 2011), or SCL (Amon & Campbell, 2008; Knox et al., 2011). *Wild Divine* included games and activities in a fantasy land with an on-screen mentor that taught breathing techniques (Bell, 2003). For example, one activity allowed children to build a bridge in order to cross a path and enter the next activity if they reached a stable pattern of breathing; if the biofeedback equipment detected an increased or unstable HR, the player was hindered from moving onto the next activity (Bell, 2003).

An additional three studies asked participants to play *Dojo*, a videogame designed to reduce anxiety in adolescents (Scholten et al., 2016; Schuurmans et al., 2015, 2018). *Dojo* was played via a computer and included HRV biofeedback and emotion regulation training through gamified contexts (Schuurmans et al., 2015). After watching instructional videos on deep breathing techniques, progressive muscle relaxation, positive thinking, and guided imagery, players were tasked with immersive puzzles to practice these strategies, which increased in difficulty if the player’s HR increased (Schuurmans et al., 2015). For instance, the “Fear Dojo” taught deep breathing techniques before the player navigated a maze to evade an angry spirit, which was only possible if their HR remained low (Schuurmans et al., 2015). All three studies evaluating *Dojo* included a trained research assistant monitoring participants to answer any questions and maintain silence if played in a group setting (Scholten et al., 2016; Schuurmans et al., 2015, 2018). Two studies evaluated user experience and found high participant satisfaction with *Dojo (*Schuurmans et al., 2015, 2018).

Participants in one study utilized *emWave*, which was also played via a computer, while completing a curriculum designed to promote emotional awareness and regulation (Rush et al., 2017). Players wore a sensor on their earlobe or finger to monitor HR, HRV, and respiratory rate. First, players were taught slow and steady breathing by pacing their breathing with a bouncing ball while being provided with immediate feedback on their HR, HRV, and respiratory rate (Rush et al., 2017). Then, children played computer games and activities to practice these skills (Rush et al., 2017). For instance, they could play a racing game where the speed of the car increased if the player had a steady rhythmic breath and decreased if the player had a faster irregular breath or quicker HR (Rush et al., 2017).

Children in two studies played *RAGE-Control* via a computer, which used HR biofeedback and was designed as an add on to anger control therapy (Ducharme et al., 2021; Kahn et al., 2013). When playing *RAGE-Control*, the child controlled a spaceship that was being attacked by aliens or asteroids (Ducharme et al., 2021; Kahn et al., 2013). If the player’s HR was too high, their ship could only fire “blanks” at the aliens or asteroids (Ducharme et al., 2021; Kahn et al., 2013). Players were rewarded for maintaining a steady HR by having the ability to control their spaceship and protect it from attacks (Ducharme et al., 2021; Kahn et al., 2013). Kahn and colleagues (2013) found high treatment compliance and player satisfaction with *RAGE-Control*.

*RAGE-Control* was later developed into *Mightier*, which continued to integrate HR biofeedback but was played via a tablet. *Mightier* is commercially available and was used in an additional two studies (Mannweiler et al., 2023; Wintner et al., 2022). Players wore an armband that monitored their HR and communicated with the *Mightier* app via Bluetooth technology (Mannweiler et al., 2023; Wintner et al., 2022). *Mightier* games included a task and an inhibitor that was activated when the player’s HR reached 7 beats per minute above their unique rolling average threshold (Mannweiler et al., 2023; Wintner et al., 2022). For example, one game asked players to slice falling fruit and avoid slicing falling trash. If the inhibitor was activated, smoke rose from the bottom of the screen and obscured the falling objects. To remove the inhibitor, players could either independently regulate their HR or engage with a guided relaxation strategy available within the game (Wintner et al., 2022). Wintner and colleagues (2022) found that most children in their sample (68%) chose to engage with *Mightier* for 5 or more weeks.

One study tasked children with using *The Pip*, which is also commercially available on iOS and Android mobile devices. Players wore a sensor on their fingers, and *The Pip* integrated skin conductance feedback (Fish, 2018). Players could visualize their baseline stress levels as well as their stress levels while gaming (Fish, 2018). In this study, use of *The Pip* was combined with a stress tracker app to assess baseline stress levels, the *Loom App* to teach players how to lower stress via visual displays (e.g., the snowy winter graphics transitioned to a vibrant summer scene with lowered SCL), and the *Relax and Race App* in which players must remain calm for their dragon to fly faster and win the race (Fish, 2018). After the intervention, teachers of students who played *The Pip* reported that the sessions were helpful to and well-received by students (Fish, 2018).

Thabrew and colleagues (2021) co-designed and evaluated *Starship Rescue*, which was also played via a tablet. *Starship Rescue* was based upon a story of a space hospital caught in a vortex of anxiety (Thabrew et al., 2021). Players were provided psychoeducation on anxiety and taught somatic relaxation and cognitive strategies, which were practiced while wearing a HR monitor that provided feedback during relaxation exercises (Thabrew et al., 2021). During the study, parents were asked to help their child choose a reward for completing the intervention, asked to validate their child’s achievement, and emailed a summary of key learning points (Thabrew et al., 2021). Multiple participants reported that they enjoyed playing *Starship Rescue* during the study (Thabrew et al., 2021).

Two studies utilized digital games with VR components. Jaramillo-Quintanar and colleagues (2020) utlized *i-Care* in their study, which was designed to manage anxiety. During play, biosensors were worn on the finger to monitor oxygen saturation and HR as proxies for feelings of anxiety (Jaramillo-Quintanar et al., 2020). First, a baseline assessment was completed to assess the players unique physiological parameters (Jaramillo-Quintanar et al., 2020). Next, an instructor taught the player diaphramatic breathing and guided imagery using visual displays of the player’s real time oxygen saturation and HR (Jaramillo-Quintanar et al., 2020). Finally, the player chose a character and designed a virtual recreational space in which they could relax (Jaramillo-Quintanar et al., 2020). During play, physiological parameters were monitored but did not appear on the screen (Jaramillo-Quintanar et al., 2020). Secondly, *Deep* was also a VR biofeedback game designed to manage anxiety and was used in an additional study (Bossenbroek et al., 2020). Players wore a belt with a stretch sensor to monitor their breathing while playing, and their respiration rate controlled their movement through an underwater fantasy world (van, 2016). Slower and deeper breaths allowed for better movement abilities around the virtual world, and there were no in game goals to achieve (Bossenbroek et al., 2020).

Lastly, Woodbury and colleagues (2021) utilized *CALMS*, which was designed to facilitate stress management and interpersonal skills within families. During the intervention, two players used handheld controllers to move spaceships throughout a screen (Woodberry et al., 2021). The goal was to deflect asteroids, which was only possible when both players’ HRs remained below their unique threshold (Woodberry et al., 2021). Researchers intermixed verbal reviews of players’ emotion regulation skills, interpersonal dynamics, and play strategies (Woodberry et al., 2021). After the intervention, children and their parents reported satisfaction with *CALMS* (Woodberry et al., 2021).

#### Targets of Interventions

Most studies evaluated internalizing symptoms or externalizing behaviors associated with mental health disorders (Amon & Campbell, 2008; Bossenbroek et al., 2020; Burkhart et al., 2018; Ducharme et al., 2021; Fish, 2018; Jaramillo-Quintanar et al., 2020; Kahn et al., 2013; Knox et al., 2011; Mannweiler et al., 2023; Rush et al., 2017; Scholten et al., 2016; Schuurmans et al., 2015, 2018; Thabrew et al., 2021; Woodberry et al., 2021). Two studies included a measure of specific skills or mechanisms of action that can contribute to the development of mental health disorders, such as emotion regulation (Mannweiler et al., 2023; Wintner et al., 2022), and four studies assessed psychopathology or functioning more generally (Amon & Campbell, 2008; Ducharme et al., 2021; Mannweiler et al., 2023; Woodberry et al., 2021. Of note, many studies included more than one outcome measure (*N* = 10; Amon & Campbell, 2008; Bossenbroek et al., 2020; Ducharme et al., 2021; Kahn et al., 2013; Knox et al., 2011; Mannweiler et al., 2023; Rush et al., 2017; Schuurmans et al., 2015, 2018; Woodberry et al., 2021). See Table 2.

The most common outcome measures evaluated internalizing symptoms. Seven studies evaluated 8 outcomes of anxiety or distress (Bossenbroek et al., 2020; Burkhart et al., 2018; Knox et al., 2011; Scholten et al., 2016; Schuurmans et al., 2015, 2018; Thabrew et al., 2021). Of these 8 anxiety-related outcomes, 7 detected statistically signficant decreases in anxiety or differences between groups, with effect sizes ranging from small to large (Bossenbroek et al., 2020; Burkhart et al., 2018; Knox et al., 2011; Scholten et al., 2016; Schuurmans et al., 2015, 2018; Thabrew et al., 2021). Four studies included an outcome related to stress or relaxation (Fish, 2018; Jaramillo-Quintanar et al., 2020; Mannweiler et al., 2023; Woodberry et al., 2021); two of which detected statistically significant improvement or differences between groups with small to large effect sizes (Mannweiler et al., 2023; Woodberry et al., 2021). Two studies included a measure of anger (Ducharme et al., 2021; Kahn et al., 2013). Kahn and colleagues (2013) detected statistically signifcant differences between groups but an effect size was not reported and not able to be determined. Lastly, one study assessed symptoms of depression and found statistically signficant differences between groups with a large effect size (Knox et al., 2011).

Six studies included a total of eight outcomes assessing externalizing behaviors (Amon & Campbell, 2008; Bossenbroek et al., 2020; Ducharme et al., 2021; Rush et al., 2017; Schuurmans et al., 2015, 2018). Four of these six studies detected statistically signficant decreases in behaviors or differences between groups (Ducharme et al., 2021; Rush et al., 2017; Schuurmans et al., 2015, 2018). Effect sizes ranged from negligible to large. Amon and Campbell (2008) measured symptoms of attention-deficit/hyperactivity disorder (ADHD; e.g., inatttention, hyperacticity, impulsivity) and found statistically signficant differences between groups with a large effect size. Last of all, Ducharme and colleagues (2021) assessed overt aggression and detected statistically signficant differences between groups with a large effect size.

Two studies included a measure of emotion regulation, which evaluated specific skills or mechanisms of action that prevent mental health disorders or signify mental health challenges (Mannweiler et al., 2023; Wintner et al., 2022). Mannweiler and colleagues (2023) found statistically significant differences in emotion regulation by treatment group with a large effect size. Wintner and colleagues (2022) also found signficant improvement in emotion regulation skills but with a small effect size.

Lastly, a quarter of the included studies assessed psychopathology or functioning more generally (Amon & Campbell, 2008; Ducharme et al., 2021; Mannweiler et al., 2023; Woodberry et al., 2021); three studies found statistcially signficant improvements or differences between groups (Amon & Campbell, 2008; Ducharme et al., 2021; Woodberry et al., 2021). Amon and Campbell (2008) found signficant differences in social, emotional, and behavioral functioning by group with a large effect size. Similarly, Ducharme and colleages (2021) identified signficant differences in psychopathology by group with a large effect size. Lastly, a feasibilty trial detected signficant improvements in social functioning with a small to medium effect size (Woodberry et al., 2021).

### Appraisal of the Quality of Studies

Of the 16 included studies, 5 were deemed randomized controlled trials (Ducharme et al., 2021; Knox et al., 2011; Mannweiler et al., 2023; Scholten et al., 2016; Schuurmans et al., 2018). Three studies were controlled trials without randomization (Amon & Campbell, 2008; Kahn et al., 2013; Rush et al., 2017). One study was a retrospective observational study (Wintner et al., 2022). The remaining 7 studies were either case studies, feasibility trials, and/or pilot studies without randomization or control (Bossenbroek et al., 2020; Burkhart et al., 2018; Fish, 2018; Jaramillo-Quintanar et al., 2020; Schuurmans et al., 2015; Thabrew et al., 2021; Woodberry et al., 2021). Of the 8 studies that included a control group, 2 studies also included blinding in their protocol (Ducharme et al., 2021; Rush et al., 2017). Five of these nine studies reported that the groups had no significant differences at baseline (Ducharme et al., 2021; Kahn et al., 2013; Mannweiler et al., 2023; Scholten et al., 2016; Schuurmans et al., 2018). All 16 studies outlined clear aims and hypotheses, documented clear benefits that outweighed risks, and considered all relevant outcomes. There was no evidence of risk of bias due to randomization, deviation from intended intervention, missing data, outcome measurement, selection of reported results, confounding, or classification of intervention. Aside from the intervention, 7 of the 8 controlled studies contained equivalent groups; the results of these 7 studies were deemed generalizable. In the excluded study, members of the treatment group had a diagnosis of ADHD, whereas members of the control group did not have a diagnosis of ADHD; thus, the results were not deemed generalizable for the purposes of this review (Amon & Campbell, 2008). See Table 2.

## Discussion

This systematic review identified 16 peer-reviewed empirical studies that evaluated biofeedback-based digital games in the treatment and prevention of youth mental health challenges. Seven of the controlled studies contained equivalent groups, so the results of these studies were deemed generalizable and are discussed in the following sections.

### Summary of Generalizable Evidence

#### Overview of Studies

Sample sizes of the 7 studies with generalizable results ranged from 24 to 138 participants (M = 52; Md = 37), participant ages ranged from 7 to 17 years old, and gender ranged from 13% to 100% female. Studies were conducted in the U.S. or the Netherlands within hospital, school, university, residential care, and community settings. All 7 studies utilized a form of cardiac biofeedback; four studies utilized HR biofeedback (Ducharme et al., 2021; Kahn et al., 2013; Mannweiler et al., 2023; Rush et al., 2017), and four studies utilized HRV biofeedback (Knox et al., 2011; Rush et al., 2017; Scholten et al., 2016; Schuurmans et al., 2018). One study also used SCL biofeedback (Knox et al., 2011), and one study also used respiratory biofeedback (Rush et al., 2017). Participants played *RAGE-Control* (Ducharme et al., 2021; Kahn et al., 2013), *Wild Divine (*Knox et al., 2011), *Mightier (*Mannweiler et al., 2023), *emWave (*Rush et al., 2017), or *Dojo (*Scholten et al., 2016; Schuurmans et al., 2018).

#### Internalizing Symptoms

Six of the studies with generalizable results included an outcome measure of internalizing symptoms. Three studies evaluated anxiety symptoms; two of which found significant decreases in anxiety symptoms with large effect sizes after playing digital games that incorporated HRV biofeedback (*Wild Divine* or *Dojo*) over 8 sessions (Knox et al., 2011; Schuurmans et al., 2018). Knox and colleagues (2011) evaluated the impact of *Wild Divine* in a sample of children with elevated anxiety. When controlling for pre-intervention scores, they found significantly lower levels of reported anxiety (and depression) in the group who played 8 sessions of *Wild Divine* than in the control group (Knox et al., 2011). Similarly, Schuurmans and colleagues (2018) evaluated the addition of *Dojo* to usual treatment at a residential care facility. After 8 30-minute sessions, both children and their mentors reported significant reductions in anxiety symptoms with a large effect size (Schuurmans et al., 2018). Alternatively, Scholten and colleagues (2016) evaluated *Dojo* with adolescents who reported elevated anxiety levels on a school-wide screener. After six 60-minute sessions, *Dojo* and the control game (*Rayman 2: The Great Escape)* both resulted in reductions of anxiety (Scholten et al., 2016), however the treatment group did have steeper decreases in personalized anxiety symptoms than the control group (Scholten et al., 2016). The differing results between these studies can possibly be explained by the differences in number of sessions or the composition of study participants. The two studies with large effect sizes recruited participants who reported elevated symptoms of anxiety (Knox et al., 2011; Schuurmans et al., 2018) or externalizing behaviors (Schuurmans et al., 2018) and included 8 treatment sessions. Alternatively, while Scholten and colleagues (2016) required participants to have elevated anxiety symptoms, they also excluded participants who were already receiving mental health care which may have resulted in a population with less severe psychopathology. In addition, participants only engaged in 6 sessions (Scholten et al., 2016).

An addditional two studies evaluated the ability of *RAGE-Control* to reduce anger via HR biofeedback (Ducharme et al., 2021; Kahn et al., 2013). Kahn and colleagues (2013) compared the impact of *RAGE-Control* to treatment as usual within an inpatient psychiatry unit and found significantly greater reductions in reported feelings of anger for children who played *RAGE-Control* for 5 30-minute sessions than children in the control group who completed treatment as usual (Kahn et al., 2013). Effect sizes were not reported and unable to be calculated. Similarly, Ducharme and colleagues (2021) compared anger control training with and without the addition of *RAGE-Control*. All children who received anger control training reported reduced experiences of angry feelings, so there were not signficant group differences (Ducharme at al. (2021). Lastly, Mannweiler and colleagues (2023) evaluated *Mightier*, an updated version of *RAGE-Control*, during summer programming within a community setting. Parents of children who played 6 30-minute sessions of *Mightier*, alongside biweekly social-emotional learning groups, reported significantly greater decreases in parenting-related stress after the intervention than parents of children in the control group (Mannweiler et al., 2023).

#### Externalizing Behaviors

Three of the studies with generalizable results evaluated externalizing behaviors (Ducharme et al., 2021; Rush et al., 2017; Schuurmans et al., 2018). In addition to anger, Ducharme and colleagues (2021) examined overt aggression and oppositional behaviors before and after anger control training with and without *RAGE-Control*, which utilizes HR biofeedback (Ducharme at al. (2021). They found significantly greater pre-post decreases in parent-reported aggression and behaviors in the group that played *RAGE-Control* compared to the group that did not play *RAGE-Control*, with large and medium effect sizes, respectively (Ducharme et al., 2021). Rush et al. (2017) evaluated the impact of *emWave*, which utilizes HR, HRV, and respiratory biofeedback, in addition to a weekly mindfulness curriculum on behaviors within special education classrooms. They found significant decreases in off-task behaviors (e.g., turning around in one’s chair, speaking about unrelated topics, looking out the window) with a large effect size after approximately 12 10-minute sessions (Rush et al., 2017). Lastly, Schuurmans and colleagues (2018) examined externalizing behaviors, in addition to anxiety, at a residential care facility. After 8 30-minute sessions of *Dojo* in addition to usual treatment they identified a significant decrease in self-reported externalizing behaviors and a non-significant change in mentor-reported externalizing behaviors compared to children who did not play *Dojo*, with medium and small effect sizes, respectively (Schuurmans et al., 2018).

#### Emotion Regulation

In addition to parent stress, Mannweiler and colleagues (2023) evaluated emotion regulation before and after playing 6 30-minute sessions of *Mightier*, which utilizes HR biofeedback, at a diverse communty summer camp. Parents of children who played *Mightier* in addition to biweekly social-emotional learning groups reported significantly greater increases in their children’s emotion regulation skills after the intervention, with a large effect size, compared to parents of children in the control group (Mannweiler et al., 2023).

#### Psychopathology/Functioning

Last of all, two of the studies with generalizable results explored psychopathology or functioning more generally (Ducharme et al., 2021; Mannweiler et al., 2023). In addition to anger, overt aggression, and oppositional behaviors, Ducharme and colleagues (2021) evaluated clinician-rated severity of psychopathology (ranging from *‘Normal*,* not ill’* to ‘*Extremely ill’*) before and after anger control training with and without *RAGE-Control*, which utilizes HR biofeedback. They found significantly greater pre-post decreases in psychopathology in the group that played *RAGE-Control* compared to the group that did not play *RAGE-Control* with a large effect size (Ducharme at al. (2021). Mannweiler and colleagues (2023) examined behaviors and feelings more generally, which included both internalizing symptoms and externalizing behaviors. They did not find signficant differences between the treatment and control groups, which may be due to the utilization of a more universal population (Mannweiler et al., 2023).

#### Strengths and Limitations

In general, biofeedback-based digital games offer promise as both a treatment and preventative intervention for a variety of youth mental health difficulties in both universal and clinical populations. Given their digital and gamified components, biofeedback-based digital games also have potential to serve as an engaging, accessible, and scalable intervention. The included studies support biofeedback-based games as a standalone treatment, as an add-on to existing mental health services, and their administration in a variety of settings. However, biofeedback-based games are a relatively new contribution to the field. To date, there are only 7 studies, with relatively small sample sizes, that offer generalizable results. Researchers have yet to extensively compare the relative efficacy of types of biofeedback or gaming platforms in this context. Also of note, there is a dearth of evidence exploring the impact of biofeedback-based games with racially and ethnically diverse populations.

Technology literacy, access to digital devices, and access to the internet are often cited concerns regarding accessibility. Recent evidence suggests that the presence of technology in the lives of American youth is all but ubiquitous (e.g., Brodsky et al., 2021; Marci, 2022). Even when examining subgroups based on race and ethnicity, parental education, and household income, no group reported less than 94% of households with technology and internet access (National Center for Educational Statistics, 2023). It is important to note that not all digital mental health interventions are created with user-centered design principles, built for diverse audiences, or well-supported by empirical evidence. Digital interventions have the potential to revolutionize mental health care access, however consideration must be taken when adopting and assessing potential interventions, especially for vulnerable populations.

Lastly, this review also contained strengths and limitations due to its design. The authors followed strict PRISMA guidelines and included a high level of double coding which enhanced the rigor of the review. In addition, studies were only included if they were published in peer-reviewed journals, which potentially increased the quality of findings but may have limited the inclusion of relevant unpublished or non-peer reviewed studies. Studies were also only included if they were available in English, and relevant studies not available in English may have been excluded due to this decision.

### Future Directions

There is a great push to incorporate mental health support within accessible settings, such as primary and secondary schools (Hoover & Bostic, 2021; Richter et al., 2022). The (Office of Disease Prevention and Health Promotion n.d.) identified increasing the proportion of youth who receive preventive mental health care within their school as a high-priority public health issue. A recent systematic review and meta-analysis found that the majority of children who access mental health services do so via their school (Duong et al., 2021). However, schools often do not have the staffing and resources to effectively provide support to all students (Eklund et al., 2017). Biofeedback-based digital games offer promise given their ability to promote childhood well-being without the resource demands of 1:1 mental health service delivery (Mannweiler et al., 2023; Rush et al., 2017; Scholten et al., 2016). We recommend that educational policymakers explore incorporating biofeedback-based digital games as a universal school-based intervention to support childhood mental health with a goal of reducing SES-related disparities in access to mental health support.

Even families with the resources to access mental health services often face lengthy wait lists (Eichstedt et al., 2024). Delayed treatment is associated with lower rates of attendance and worse clinical outcomes (Diego-Adeliño et al., 2010; Sherman et al., 2009; Williams et al., 2008). Biofeedback-based digital games offer promise as an interim treatment while children are waiting for treatment (Knox et al., 2011). Mental health providers should explore adding biofeedback-based digital games as an option for families on their waitlist for treatment. Additionally, biofeedback-based digital games have shown promise as an add-on treatment to existing mental health therapies, especially within higher levels of care (Ducharme et al., 2021; Kahn et al., 2013; Schuurmans et al., 2018). Mental health providers in such settings should actively explore ways to augment existing treatments with biofeedback-based digital games, and researchers should explore the augmentation of outpatient mental health services with biofeedback-based digital games to reach even more children and their families.

Lastly, biofeedback-based digital interventions have the ability to be utilized at home (Wintner et al., 2022) which may offer a viable strategy to increase accessibility of services for families who face transportation barriers or live in rural communities. The included studies did not evaluate the specific ability of biofeedback-based digital interventions to increase accessibility for such families; this is an important area for future research. Additionally, some biofeedback-based digital interventions are commercially available for in-home use, but they can be costly and out of reach for families that rely on public or private health insurance to access mental health services. A third of children in the U.S. receive medical insurance coverage via Medicaid, a government funded program that provides health insurance to families of low-income in the U.S. (37%; Assistant Secretary for Public Affairs, 2023; United States Census Bureau, 2022). An additional 62% of children have private health insurance (United States Census Bureau, 2022). The vast majority of families use their public or private health coverage to pay for their children’s mental health services (Kemp, 2024). Thus, insurance providers, researchers, and mental health professionals should explore the cost-benefits of medical insurance coverage for biofeedback-based digital games.

## Conclusion

Biofeedback-based digital games offer one promising solution to address the burgeoning youth mental health crisis. Sixteen studies were identified in this systematic review, seven of which were controlled studies that evaluated equivalent groups aside from the intervention and, thus, elicited generalizable results. The 5 digital games with the most promising results were *RAGE-Control*,* Wild Divine*,* Mightier*,* emWave*, and *Dojo*, all of which utilize a form of cardiac biofeedback (HR or HRV). They were evaluated in a variety of settings as add-on and standalone treatments with both universal and clinical populations. These games proved effective in reducing anger, anxiety, depression, stress, aggression, externalizing behaviors, and general psychopathology, especially within samples of children who had elevated levels of symptomology at baseline. In addition, there is promising evidence of their ability to promote emotion regulation skills in more universal populations. Future directions include further evaluation in school-based settings, as an interim outpatient treatment while children are waiting to see mental health providers, as an add-on to existing evidence-based treatments, and of the cost-benefits of insurance coverage for biofeedback-based digital interventions.

## Data Availability

All relevant datasets are available from the corresponding author upon request.
